# Changes in ruminal lipopolysaccharide, fermentation characteristics, nutrient digestibility, blood variables, and microbiota in sheep with subacute ruminal acidosis and its regulation by polymyxin CP7

**DOI:** 10.1016/j.vas.2026.100878

**Published:** 2026-09-23

**Authors:** Shuai Jiao, Baozhen Zhu, Fei Li, Zhiyuan Ma, Fadi Li, Xiuxiu Weng, Ying Yu, Ping Li, Rui Xu

**Affiliations:** a State Key Laboratory of Grassland Agro-ecosystems, Key Laboratory of Grassland Livestock Industry Innovation, Ministry of Agriculture and Rural Affairs, Engineering Research Center of Grassland Industry, Ministry of Education, College of Pastoral Agriculture Science and Technology, Lanzhou University, Lanzhou, 730020, China; b Gansu Olymbel Biotechnology Co., Ltd, Zhangye, 734000, China; c Gansu Kunlun Biochemical Co., Ltd, Zhangye, 734000, China

**Keywords:** Polymyxin CP7, Lipopolysaccharide, Rumen fermentation, Blood index, Microflora

## Abstract

The objectives of this study were to (1) determine the effects of induced subacute ruminal acidosis (SARA) on ruminal lipopolysaccharide (LPS), nutrient apparent digestibility, ruminal fermentation parameters, hematological indices, and ruminal microbiota; and (2) evaluate the responses associated with polymyxin CP7 supplementation during SARA in Hu sheep. Eight rumen-fistulated Hu sheep sequentially underwent three 29-day stages: a control stage (CON, basal diet), a SARA induction stage (SAR, high-barley diet), and a bacteriocin-supplemented stage (BAC, high-barley diet with 500 mg/kg polymyxin CP7). The sequential design was adopted because SARA is not immediately reversible, rendering crossover designs problematic due to potential carry-over effects. Rumen pH remained below 5.60 for 315 min/d during the SAR stage, fulfilling the diagnostic criteria for SARA. Ruminal LPS concentration in the SAR stage was higher than that in the CON and BAC stages (*P* < 0.05). The apparent digestibility of organic matter and neutral detergent fiber in the BAC stage was higher than that in the CON and SAR stages, whereas dry matter intake was lower than that in the CON stage (*P* < 0.05). The propionate proportion was higher in the BAC stage than in the CON and SAR stages (*P* = 0.005). The butyrate proportion and lactic acid concentration in the SAR stage were higher than that in the other two stages (*P* < 0.05). Red blood cell hemoglobin and mean red blood cell volume in the SAR stage were lower than those in the CON and BAC stages (*P* < 0.05), whereas mean red blood cell hemoglobin concentration, platelet, and platelet hematocrit were higher in the SAR stage than in the other two stages (*P* < 0.01). Additionally, the monocyte proportion in the BAC stage was higher than that in the CON and SAR stages (*P* < 0.01). The relative abundance of the genus *Moryella* in the SAR stage was higher than that in the CON and BAC stages (*P* < 0.05), whereas the relative abundance of the genus *Treponema* in the BAC stage was lower than that in the CON and SAR stages (*P* < 0.05). In conclusion, although ruminal acidosis persisted during the BAC stage, lower ruminal LPS concentration, higher nutrient digestibility, and alterations in ruminal fermentation and microbial composition were observed during this stage. These findings suggest that polymyxin CP7 may mitigate selected SARA-associated responses.

## Highlights

• Ruminal LPS was lower in BAC than SAR while acidosis persisted.
• OM and NDF digestibility were higher in BAC than in CON and SAR.
• BAC showed higher propionate proportion and lower lactate concentration than SAR.
• Monocyte proportion was higher in BAC than in CON and SAR.
• Relative abundances of *Moryella* and *Treponema* were lower in BAC than SAR.

## 1 Introduction

In the production of fattening sheep, high-grain diets are commonly employed as the primary feeding strategy to meet energy requirements and enhance productivity (Bo Trabi et al., 2019). However, the rapid fermentation rate and high digestibility of grains in the rumen result in an increase in the concentration of volatile fatty acids (VFA) and a decrease in rumen pH, leading to the development of subacute rumen acidosis (SARA) and subsequently reducing dry matter intake (DMI) and production performance (Yusuf et al., 2017). The subacute ruminal acidosis has been defined as a decline in ruminal pH < 5.6 for more than 180 min per day in dairy cow (AlZahal et al., 2007; Plaizier et al., 2008). Low rumen pH leads to the lysis of Gram-negative bacteria and the accumulation of lipopolysaccharide (LPS) in the rumen (Li et al., 2012). Analogously, Gozho et al. (2005) showed that ruminal LPS concentration increased significantly during periods of grain feeding compared with times when only hay was fed. Further research suggests induction of SARA elevated serum amyloid A concentration from 286.8 to 498.8 g/mL in mid lactation dairy cows, but did not affect other markers of inflammation including haptoglobin, fibrinogen, serum copper, or white blood cells (Gozho et al., 2007). In contrast, increased LPS content improves white blood cell counts in the blood and induces innate immune responses (Ramadan et al., 2020). These inconsistent findings suggest that systemic inflammatory responses to SARA may vary depending on the severity and duration of the acidotic challenge, animal physiological status, and the specific inflammatory markers evaluated. Taken together, grain-induced SARA increases the cleavage of Gram-negative bacteria, LPS accumulates in the rumen, and activates the inflammatory response. Therefore, the search for novel additives that can mitigate the release of LPS and alleviate the detrimental effects of SARA is of utmost importance in livestock production.

With the prohibition of antibiotics, bacteriocins, a promising alternative to antibiotics, can be used as feed additives in animal production to protect livestock from pathogen damage. Bacteriocins are a class of small, heat-stable, ribosomally synthesized antimicrobial peptides that are produced by bacteria and archaea (Dobson et al., 2012). They are active against other bacteria, and the producers are immune to them (Dobson et al., 2012). Bacteriocins employ various mechanisms to exert their antimicrobial activity, inducing cytoplasmic membrane permeability through pore formation, inhibiting cell wall biosynthesis, and interfering with metabolic pathways (Darbandi et al., 2022). In terms of probiotic properties, bacteriocins act as colonization peptides, facilitating the survival of production strains in the intestinal environment (Walsh et al., 2008). Additionally, bacteriocins play a growth-promoting role by directly inhibiting the growth of pathogens (Corr et al., 2007). Finally, bacteriocins can function as signal peptides in the intestinal environment (Van Hemert et al., 2010).

Polymyxin E1 isolated from *Paenibacillus polymyxa* has significant antibacterial activity against Pseudomonas and Escherichia coli, and mainly acts on LPS of the outer membrane (Velkov et al., 2010). Similarly, *Paenibacillus polymyxa JB-0501* inhibited the growth of E. coli ATCC 25922, as proven by the diffusion tests in agar, thus had the potential probiotic properties to justify its consideration as a livestock feed supplement (Naghmouchi et al., 2013). In this study, polymyxin CP7 is a bioactive peptide isolated from *paenibacillus polymyxa CP7*. There are various indications that polymyxin CP7 has the potential to alleviate LPS accumulation by inhibiting the activity of pathogenic bacteria such as *Escherichia coli*. Therefore, we hypothesized that dietary supplementation with polymyxin CP7 could mitigate selected SARA-associated responses and reduce ruminal LPS concentration. Accordingly, we investigated the effects of polymyxin CP7 on ruminal LPS, ruminal fermentation, microbiota, hematological indices, and apparent nutrient digestibility in Hu sheep with induced SARA.

## 2 Materials and methods

The experimental protocol was approved by the Animal Ethics Committee of Lanzhou University, and humane animal care and handling procedures were implemented throughout the experiment.

### 2.1 Animal experimental design

Eight castrated *Hu* sheep fitted with rumen fistulas and weighing 53.78 ± 3.14 kg were used in the study. All sheep sequentially underwent three experimental stages: a control stage (CON), a SARA induction stage (SAR), and a bacteriocin supplementation stage (BAC). Each stage lasted 29 days, consisting of a 7-day transition period followed by a 22-day experimental period (Fig. 1). During the CON stage, sheep received the basal diet. During the SAR stage, sheep received a high-barley diet containing 25% finely ground barley to induce SARA. During the BAC stage, sheep continued to receive the same high-barley diet as in the SAR stage, with the addition of 500 mg/kg polymyxin CP7. Polymyxin CP7 was added directly to the diet without replacing any other dietary ingredient. The dietary composition of the three stages is presented in Table 1. The polymyxin CP7 used in this study was a purified peptide with a purity of 98% and was supplied by Gansu Orinbel Biotechnology Group Co., Ltd. (Lanzhou, China). It was directly incorporated into the feed ingredients before pelleting at 500 mg/kg diet. The three stages were conducted sequentially in the order CON, SAR, and BAC. This sequential design was adopted because SARA may not be immediately reversible, which could introduce carry-over effects in alternative designs such as a Latin square.

**Fig. 1:**
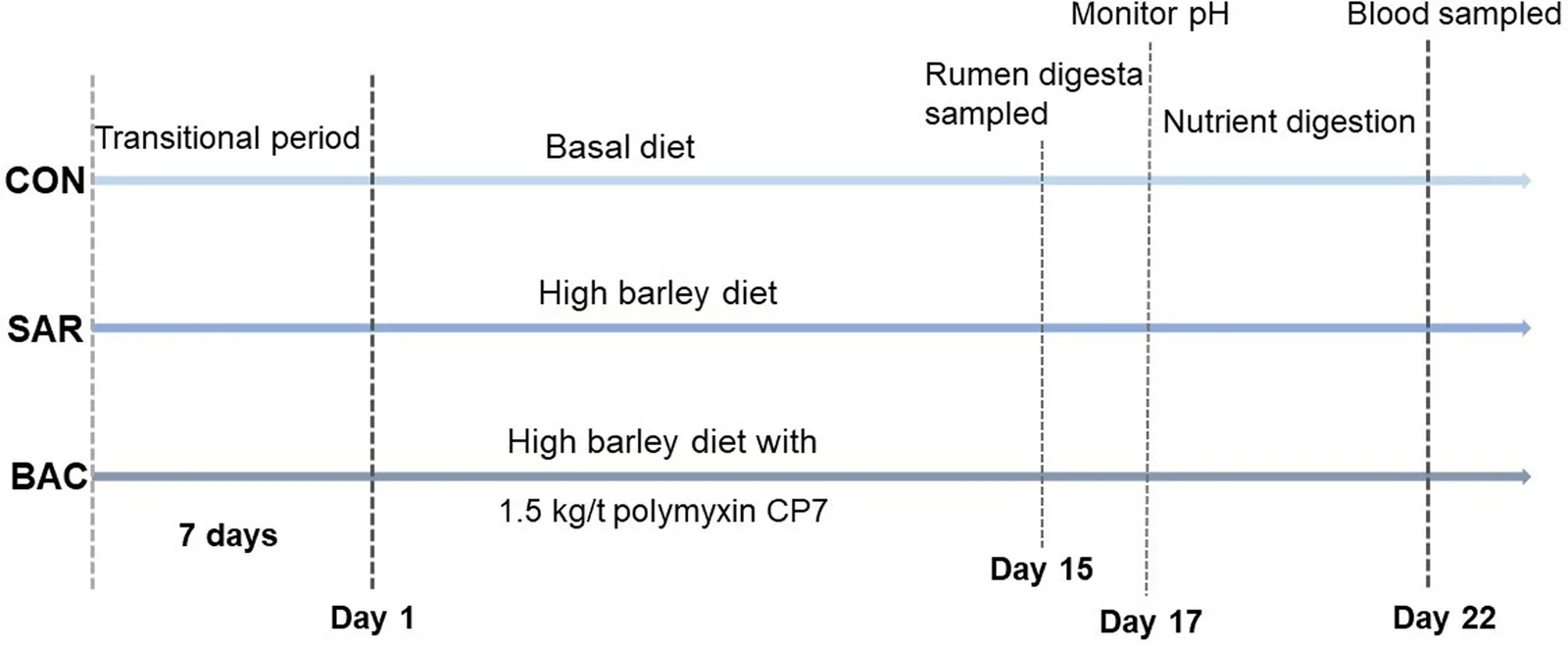
Diagram of experimental design. CON= control stage (fed basal diet); SAR= SARA induction stage (fed high barley diet); BAC= bacteriocin feeding stage (fed high barley diet with 500 mg/kg polymyxin CP7).

**Table 1:** Composition and nutritional levels of diets (% as DM basis).

Items	Content^b^		
	CON	SAR	BAC
Ingredients			
Corn straw	25.00	25.00	25.00
Corn	26.40	26.40	26.40
Barley	0.00	25.00	25.00
Corn bran	25.00	0.00	0.00
Molasses	2.00	2.00	2.00
Soybean meal	5.28	5.28	5.28
Cottonseed meal	5.28	5.28	5.28
Corn germ feed	8.93	8.93	8.93
Limestone	0.86	0.86	0.86
NaCl	0.48	0.48	0.48
Expanded Urea	0.38	0.38	0.38
Premix^a^	0.38	0.38	0.38
Total	100.00	100.00	100.00
Nutritional levels, %DM			
Dry matter	90.25	89.49	89.40
Crude protein	14.35	14.85	15.26
Neutral detergent fiber	39.68	28.15	26.84
Acid detergent fiber	16.31	13.98	12.85
Ether extract	2.43	2.06	2.10
Starch	17.74	27.87	27.95

a The premix provided per kg of diets: Fe, 25 mg; Zn, 40 mg; Cu, 8 mg; Mn, 40 mg; I, 0.3 mg; Se, 0.2 mg; Co, 0.1 mg; vitamin A, 940 IU; vitamin D, 111 IU; vitamin E, 20 IU.

b CON = control stage (fed basal diet); SAR = SARA induction stage (fed high barley diet); BAC = bacteriocin feeding stage (fed high barley diet with 500 mg/kg polymyxin CP7).

### 2.2 Feeding and management

During the experiment, all sheep were raised in a single pen which was 1.8 m × 1.25 m × 1 m. All sheep were fed twice at 0700 h and 1800 h and were allowed free access to water. The feed offered and residual feed of each sheep were accurately recorded daily in order to determine their DMI.

### 2.3 Rumen pH and LPS

The rumen pH of all sheep was continuously monitored for 24 h on days 17–18 of the trial period. The rumen pH monitoring system consists of a pH electrode (model IP-600-10, JENCO, Shanghai Ren Electronics Co., LTD.), a pH transmitter (model 691, JENCO, Shanghai Ren Electronics Co., LTD.) and a paperless recorder (model ZDR-wn, Hangzhou Zeda Instrument Co., LTD.). Prior to the morning feeding, the pH electrode was calibrated using standard pH solutions of 4.0 and 7.0. The electrode was then inserted into the rumen via the rumen fistula. Data were recorded every 0.5 min to capture real-time changes in rumen pH. Based on the recorded data, the mean rumen pH value, as well as the duration and extent of pH below 5.80 and 5.60, were calculated. The acidosis index was calculated as the area of ruminal pH below 5.80 divided by DMI (pH × min/kg DMI), thereby normalizing the magnitude and duration of ruminal pH depression for feed intake. This index has previously been used to characterize individual variation in SARA severity in ruminants (Zhang et al., 2023).

Rumen contents were collected and filtered at 0, 2, 4, 6, 8 and 12 h after the morning feeding on days 15–16 of the trial period. The collected rumen fluid was stored in a -80°C refrigerator for subsequent determination of LPS. The determination of LPS was performed using the chromogenic substrate-specific Limulus test kit (Xiamen Limulus Reagent Biotechnology Co., LTD., Xiamen), and the absorbance values were measured at a wavelength of 545 nm using a model 721 Spectrophotometer.

### 2.4 Apparent digestibility of nutrients

The digestion test was conducted from day 17 to day 21 of the experimental period. During this period, total feces were collected at 8:00 a.m. every day and fecal output was recorded. Approximately 10% of the total weight of feces was gathered, placed into an aluminum box, and subjected to drying at 65°C. Furthermore, about 1% of fresh fecal samples were transferred into 500 mL brown jars, followed by the addition of a 10% sulfuric acid solution, ensuring complete immersion of the fecal samples. The fecal samples from the 5-day collection period were amalgamated into mixed samples, lyophilized, and pulverized for the determination of crude protein (CP) content. Feed intake and refusal were recorded daily throughout this duration. The DM, CP, NDF, and ADF in the pelleted feeds, residual pelleted feeds and feces were measured according to AOAC (2005).

### 2.5 Fermentation parameters

The rumen digesta was collected on day 15 of the trial period and filtered through four layers of gauze. Then, 250 g/L metaphosphate was added. The collected rumen fluid was stored in a -80°C refrigerator for subsequent determination of VFA concentrations. VFA in the rumen fluid were determined using a gas chromatograph (Themo Scientific TRACE 1300, Italy) equipped with the DP-FFAP capillary chromatographic column. The injection volume was 1 μL, and the split ratio was 50:1. The concentration of ammonia nitrogen was determined using the phenol-sodium hypochlorite colorimetric method. The lactic acid content of the rumen fluid was determined using the lactic acid assay kit (A019-2-1, Nanjing Institute of Jiancheng Bioengineering, Nanjing).

### 2.6 Blood index

Two blood samples were collected from the jugular vein before morning feeding on day 22 of each stage. One part was transferred into heparin sodium tube (Heze Huabo Medical Products Co., Ltd., China), centrifuged at 4°C (3000 × g, 15 min) to obtain plasma, and frozen at -80°C for the determination of plasma glucose (GLU) and urea nitrogen (BUN). The content of GLU (glucose oxidase method, Shanghai Rongsheng Biotechnology Co., LTD.) and BUN (urease method, Nanjing Jiancheng Technology Co., LTD.) was determined by kit method. The other part is used to measure blood routine. The blood cell composition was immediately determined by an automated blood cell analyzer (ProCyte Dx®, USA).

### 2.7 Bacteria 16S rDNA sequencing

The bacterial DNA of 24 samples (8 samples per stage) was extracted, and the specific primers with Barcode were synthesized according to the 16S full-length primers 27F and 1492R. After that, PCR amplification was performed and the products were purified, quantified and homogenized to form a sequencing library. The constructed library was first subjected to library quality inspection, and the qualified library was sequenced by PacBio Sequel. Based on the PacBio Sequencing platform, the marker gene was sequenced by single-molecule real-time sequencing method, and then Optimization-CCS was obtained by filtering the sequence of Circular Consensus Sequencing (CCS). The Usearch software was used to cluster Tags at 97% similarity level and obtain Operational Taxonomic Units (OTUs) (Edgar, 2013). After the taxonomic annotation of OTUs, QIIME software was used to generate species abundance at different levels. Moreover, the α-diversity of microflora was analyzed based on OTU, and the flora structure was analyzed at the taxonomic level of phylum and genus.

### 2.8 Statistical analysis

Data on dry matter intake, apparent nutrient digestibility, ruminal fermentation characteristics, blood parameters, and microbial diversity indices were analyzed using a mixed model procedure in SAS procedure. Because the same animals were evaluated sequentially across the three experimental stages, experimental stage (CON, SAR, and BAC) was included as a fixed effect and animal was included as a random effect to account for between-animal variability: Y_ij_= μ+ S_i_+ A_j_+ ε_ij_, where Y_ij_ is the dependent variable, μ is the overall mean, S_i_ is the fixed effect of experimental stage, A_j_ is the random effect of animal, and ε_ij_ is the residual error. Because all animals underwent the experimental stages in the same order, experimental stage was completely confounded with chronological period. Consequently, the model evaluates differences among experimental stages but does not allow treatment effects to be statistically separated from potential period, adaptation, or carry-over effects. Comparisons involving the BAC stage were therefore interpreted cautiously.

Ruminal LPS concentrations collected at multiple sampling times were analyzed as repeated measures using a mixed model with experimental stage, sampling time, and their interaction as fixed effects, and animal as a random effect: Y_ijk_= μ+ S_i_+ A_j_+ H_k_+ (S × H)_ik_+ ε_ijk_, where H_k_ represents the effect of sampling time, (S × H)_ik_ represents the interaction between experimental stage and sampling time, and ε_ijk_= the random residual error. When significant effects were detected, means were separated using Duncan’s multiple range test. Differences were considered significant at *P* < 0.05.

Alpha-diversity indices were used to evaluate within-sample microbial diversity. Differences in microbial community structure among the experimental stages were visualized using principal coordinate analysis (PCoA), and statistical significance was evaluated using ANOSIM. LEfSe analysis was used as an exploratory approach to identify taxa with differential relative abundance among the experimental stages.

## 3 Results

### 3.1 Ruminal pH

The mean and minimum values of rumen pH in SAR and BAC were lower than those in CON (*P* < 0.01, Table 2). The duration time of pH < 5.60 or 5.80 in SAR and BAC was higher than that in CON (*P*= 0.003). The area under pH 5.80 in SAR and BAC were higher than that in CON (*P* < 0.01). DMI in BAC was lower than that in CON (*P* < 0.05). However, the acidosis index was the highest in BAC, followed by the SAR, and the lowest in CON (*P* < 0.01).

**Table 2:** The variations of DM intake and ruminal pH in three stages.

Items	Treatment^a^			SEM	P-value^b^
	CON	SAR	BAC		
DM intake, kg/d	1.66^a^	1.44^ab^	1.27^b^	0.133	0.031
Mean	6.49^a^	5.90^b^	5.93^b^	0.084	0.001
Maximum	7.16	7.03	7.44	0.074	0.064
Minimum	5.80^a^	5.15^b^	4.95^b^	0.118	0.003
Duration time of ruminal pH, min/d					
pH < 5.80	84.25^b^	659.00^a^	630.57^a^	79.812	0.001
pH < 5.60	47.38^b^	315.38^a^	482.29^a^	60.568	0.007
Area under curve, pH × min/d					
pH < 5.80	21.18^b^	157.62^a^	311.32^a^	46.491	0.034
pH < 5.60	8.96	61.07	199.82	35.250	0.076
Acidosis index, pH × min/kg	12.89^c^	110.20^b^	263.53^a^	27.85	<0.001

a CON = control stage (fed basal diet); SAR = SARA induction stage (fed high barley diet); BAC = bacteriocin feeding stage (fed high barley diet with 500 mg/kg polymyxin CP7).

b Within a row, means without a common superscript letter differ at *P* < 0.05.

### 3.2 Ruminal LPS

To investigate the impact of SARA induction and bacteriocin supplementation on ruminal endotoxin load, diurnal changes in ruminal LPS concentration were monitored at 0, 2, 4, 6, 8, and 12 h after feeding throughout the three experimental stages. As illustrated in Fig. 2, ruminal LPS concentration in the SAR stage remained significantly higher than that in the CON and BAC stages across all monitored time points (*P* < 0.01).

**Fig. 2:**
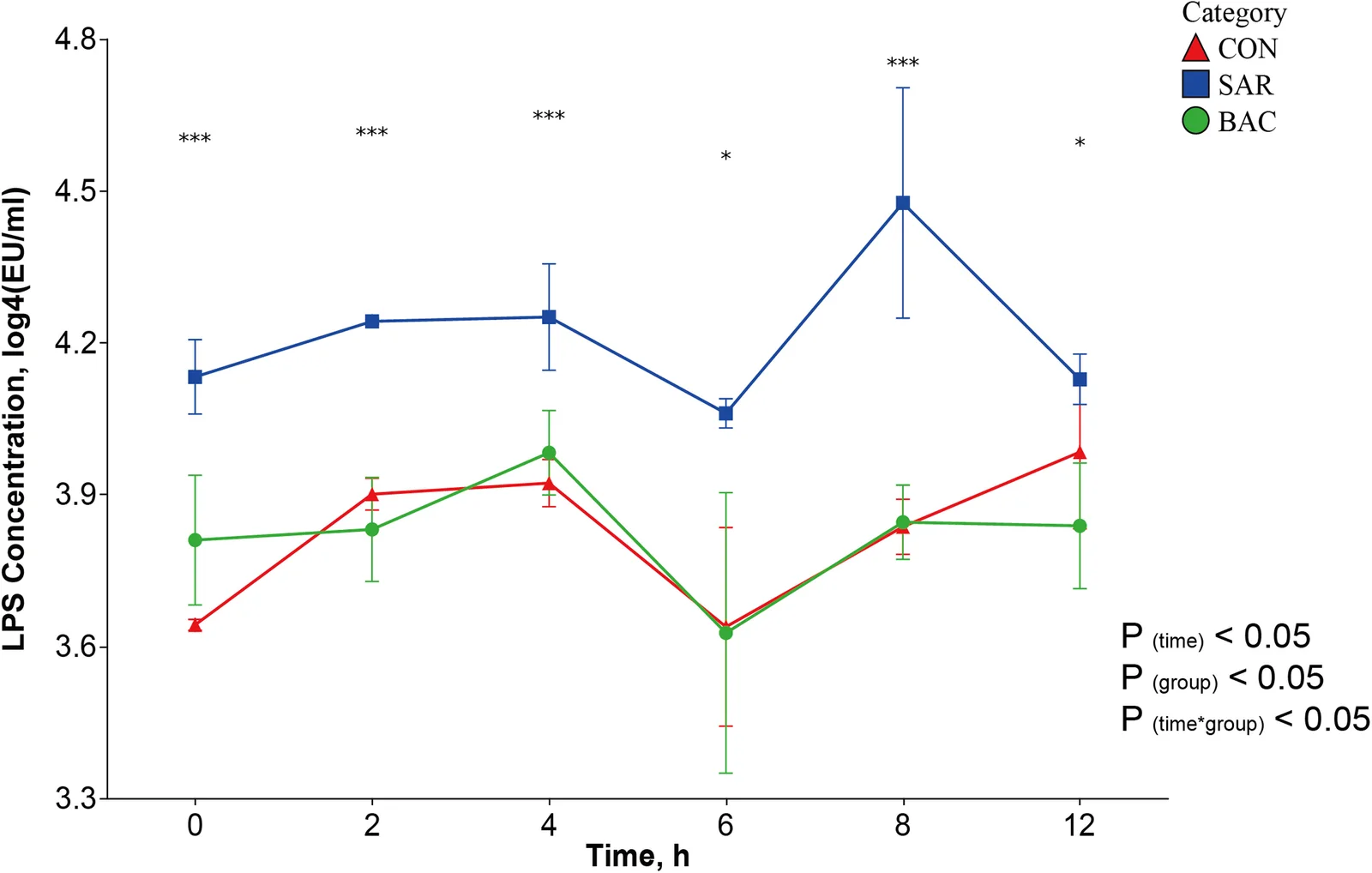
Effects of different stages on rumen dynamic concentration of LPS. CON= control stage (fed basal diet); SAR= SARA induction stage (fed high barley diet); BAC= bacteriocin feeding stage (fed high barley diet with 500 mg/kg polymyxin CP7). *P < 0.05, **P < 0.01, ***P < 0.001.

### 3.3 Nutrient digestion and fermentation parameters

The apparent digestibility of OM and NDF in BAC were higher than those in CON and SAR (*P* < 0.05, Table 3). There was no significant difference in the apparent digestibility of DM, CP and ADF among the three treatment stages (*P* > 0.05).

**Table 3:** Effects of different stages on nutrient digestion and fermentation parameters of *Hu* lambs.

Items	Treatment^a^			SEM	P-value^b^
	CON	SAR	BAC		
Apparent digestibility, %					
DM	66.96	66.37	67.29	1.107	0.925
OM	68.34^b^	67.30^b^	72.17^a^	0.853	0.023
CP	72.05	69.91	70.14	0.825	0.504
NDF	50.54^b^	51.35^b^	58.31^a^	0.943	0.001
ADF	35.72	30.57	37.11	1.211	0.070
Fermentation parameters					
NH_3_-N, mmol/L	0.80^b^	1.67^a^	1.38^a^	0.074	<0.001
TVFA, mmol/L	79.78	82.63	87.61	2.382	0.408
Acetate, %	57.65^a^	54.84^b^	54.27^b^	0.514	0.015
Propionate, %	25.33^b^	24.26^b^	28.83^a^	0.606	0.005
Isobutyrate, %	0.52^b^	1.01^a^	1.04^a^	0.065	0.001
Butyrate, %	14.63^b^	16.83^a^	13.44^b^	0.359	<0.001
Isovaleate, %	0.66^b^	1.42^a^	1.35^a^	0.089	<0.001
Valerate. %	1.21^b^	1.64^a^	1.55^a^	0.042	<0.001
Acetate/Propionate	2.38	2.49	2.22	0.086	0.439
Lactic acids, mmol/L	0.83^b^	1.34^a^	0.84^b^	0.078	0.026

a CON = control stage (fed basal diet); SAR = SARA induction stage (fed high barley diet); BAC = bacteriocin feeding stage (fed high barley diet with 500 mg/kg polymyxin CP7).

b Within a row, means without a common superscript letter differ at *P* < 0.05.

The concentration of NH_3_-N in the CON was lower than that in other treatment stages (*P* < 0.001, Table 3). Ruminal TVFA concentration was not significantly different among treatment stages (*P* > 0.05). The proportion of acetate in SAR and BAC was lower than that in CON (*P*= 0.015), but the proportion of isobutyrate, isovaleate and valerate in SAR and BAC was higher than that in CON (*P* < 0.01). The propionate proportion was higher in the BAC stage than in the CON and SAR stages (*P*= 0.005). The butyrate proportion and lactic acid concentration in the SAR stage was higher than that in the other two stages (*P* < 0.05). The acetate/propionate ratio was not significantly different among treatment stages (*P* > 0.05).

### 3.4 Blood index

The red blood cell hemoglobin (RBC-He) content and mean red blood cell volume (MCV) in SAR were lower than those in CON and BAC (*P* < 0.05, Table 4), while the mean red blood cell hemoglobin concentration (MCHC), platelet (PLT) and platelet hematocrit (PCT) were higher than those in the other two stages (*P* < 0.01). The PLT and PCT in BAC were lower than those in CON and SAR (*P* < 0.001). In addition, the proportion of monocyte (MONO) in BAC was higher than that in CON and SAR (*P* < 0.01). The lymphocyte (LYMPH) in BAC was lower than that in CON (*P* < 0.05). There were no significant differences in other blood routine indexes and urea nitrogen content among different stages (*P* > 0.05).

**Table 4:** Effects of different stages on blood index of *Hu* lambs.

Items^a^	Treatment^b^			SEM	P-value^c^
	CON	SAR	BAC		
Red blood cells and hemoglobin					
RBC(M/μL)	14.38	15.19	14.88	0.246	0.433
HCT(%)	38.69	33.39	39.76	1.217	0.059
HGB(g/dL)	13.79	14.38	14.08	0.231	0.650
RBC-He(pg)	11.53^a^	10.74^b^	11.53^a^	0.132	0.010
MCV(fL)	26.99^a^	22.03^b^	26.83^a^	0.835	0.013
MCH(pg)	9.60	9.46	9.49	0.132	0.914
MCHC(g/dL)	35.66^b^	43.55^a^	36.06^b^	1.165	0.003
RDW-SD(fL)	27.20	27.10	27.64	0.480	0.894
RDW-CV(%)	41.70	42.26	42.45	0.522	0.849
Blood platelet					
PLT(K/μL)	341^b^	478^a^	111^c^	36.515	<0.001
MPV(fL)	8.76	8.34	8.16	0.108	0.069
PCT(%)	0.30^b^	0.40^a^	0.09^c^	0.031	<0.001
Blood immune cells					
WBC(K/μL)	8.57	11.63	7.92	1.141	0.372
NEUT(K/μL)	2.72	6.19	3.05	1.131	0.399
LYMPH(K/μL)	5.09^a^	4.36^ab^	3.55^b^	0.260	0.048
MONO(K/μL)	0.47	0.77	1.04	0.108	0.099
EO(K/μL)	0.25	0.26	0.24	0.055	0.987
NEUT(%)	31.34	41.03	37.33	3.048	0.458
LYMPH(%)	59.77	48.43	45.66	3.126	0.163
MONO(%)	5.10^b^	7.11^b^	13.21^a^	1.379	0.002
EO(%)	3.24	2.85	3.26	0.673	0.963
BASO(%)	0.54	0.59	0.54	0.075	0.959
Blood biochemical indexes					
BUN, mmol/L	7.64	7.36	6.95	0.348	0.737
GLU, mmol/L	4.99	4.72	4.42	0.106	0.088

a Abbreviations: BASO = Basophils; BUN = Urea nitrogen; EO = Eosinophils; GLU = Glucose; HCT = Hematocrit; HGB = Hemoglobin; LYMPH = Lymphocytes; MCH = Mean red blood cell hemoglobin; MCHC = Mean red blood cell hemoglobin concentration; MCV = Mean red blood cell volume; MONO = Monocyte; MPV = Mean platelet volume; NEUT = Neutrophils; PCT = Platelet hematocrit; PLT = Platelet; RBC = Red blood cells; RBC-He = Red blood cell hemoglobin; RDW-CV = Coefficient of variation of erythrocyte distribution width; RDW-SD = Standard deviation of red blood cell distribution width; WBC = White blood cell.

b CON = control stage (fed basal diet); SAR = SARA induction stage (fed high barley diet); BAC = bacteriocin feeding stage (fed high barley diet with 500 mg/kg polymyxin CP7).

c Within a row, means without a common superscript letter differ at *P* < 0.05.

### 3.5 Ruminal microorganism

Full-length 16S rRNA genes were amplified using primers 27F and 1492R and sequenced on the PacBio Sequel platform. After sequencing and sequence filtering of 24 samples, 147934 Optimization-CCS were obtained for analysis. Based on the principle of similarity greater than 97%, the effective sequences were clustered and the chimeras were filtered to obtain a total of 361 OTUs for species classification. 252 OTUs were shared among the three stages (Fig. 3B). The number of OTUs in the CON, SAR and BAC stages were 298, 326 and 314, respectively. ACE, Chao1, Simpson and Shannon index did not differ among the three stages (*P* > 0.5, Fig. 3A). PCoA revealed differences in microbial community structure among the experimental stages, which were further supported by ANOSIM (P= 0.003, Fig. 3C and D).

**Fig. 3:**
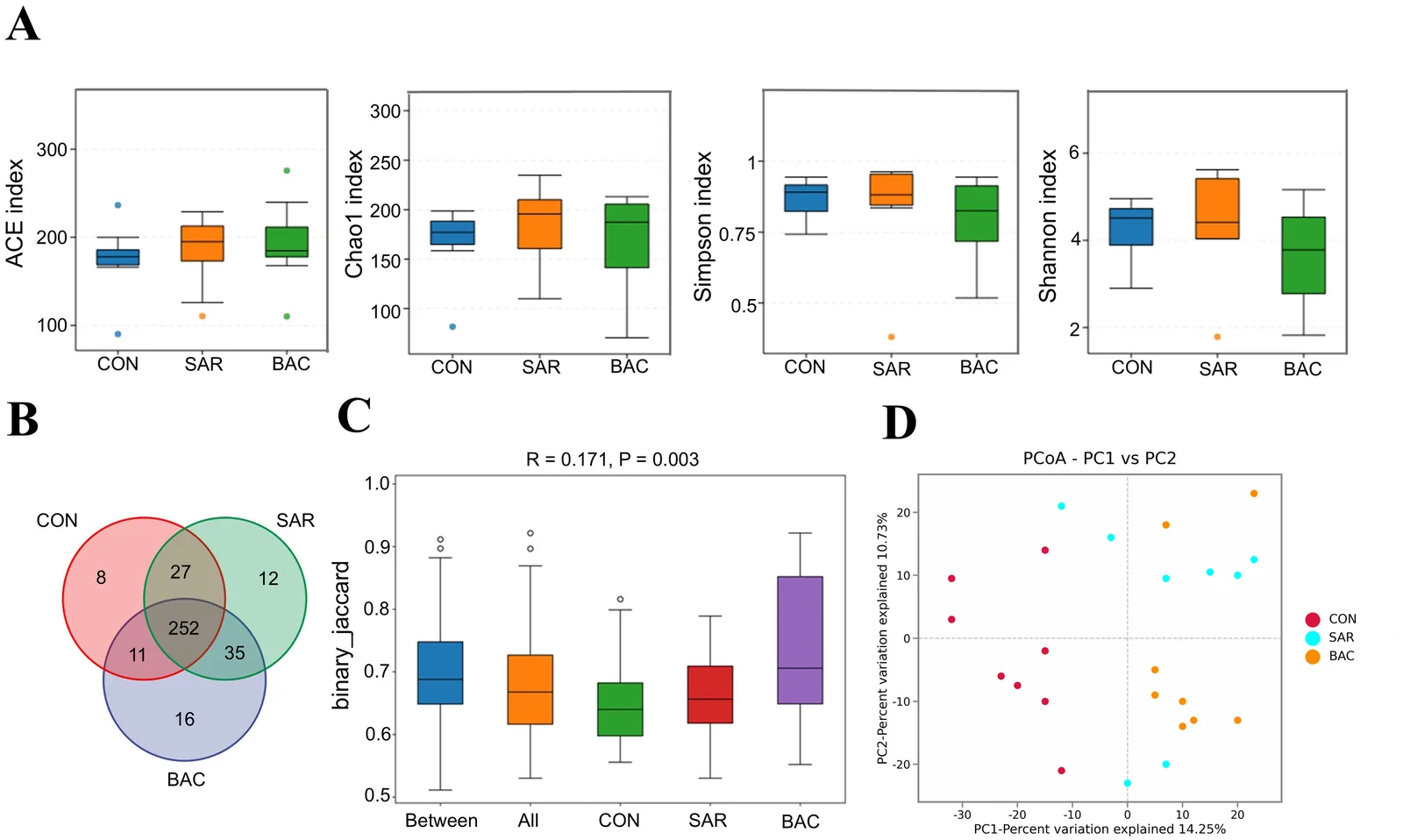
Effects of different stages on rumen microbial diversity of *Hu* lambs. A. alpha diversity. B. OTU Venn Diagram. C. Anosim analysis based on binary_jaccard algorithm. D. PCoA diagram of among stages. CON= control stage (fed basal diet); SAR= SARA induction stage (fed high barley diet); BAC= bacteriocin feeding stage (fed high barley diet with 500 mg/kg polymyxin CP7).

In terms of microbial composition, the dominant bacteria at the phylum level were *Firmicutes, Bacteroidetes* and *Proteobacteria*, which accounted for more than 90 % of the total abundance (Fig. 4A). The top twenty relative abundances at genus level were *Prevotella, Quinella, Bact, Ruminococcus, Succiniclasticum*, etc (Fig. 4B). To confirm the differences of bacteria among stages, LEfSe was carried out for taxon at all levels to find the featured genera among stages. Phylum *Verrucomicrobiota* was the biomarkers in SAR stage, and genera *Clostridium* was the biomarkers in BAC stage. Phylum *Spirochactales*, and genera *Victivallis, Treponema* were featured bacteria for CON stage (Fig. 5A). The relative abundance of genus *Moryella* in the SAR stage was higher than that in the CON and BAC stage (*P* < 0.05, Fig. 5B). *Prevotella* was the genus with the highest relative abundance, but there was no significant difference in its abundance at each stage (*P* > 0.05). In addition, the relative abundance of *Clostridium* in the SAR and BAC was higher than that in the CON stage (*P* < 0.05), and the relative abundance of *Treponema* in the CON and SAR stage was higher than that in the BAC stage (*P* < 0.05).

**Fig. 4:**
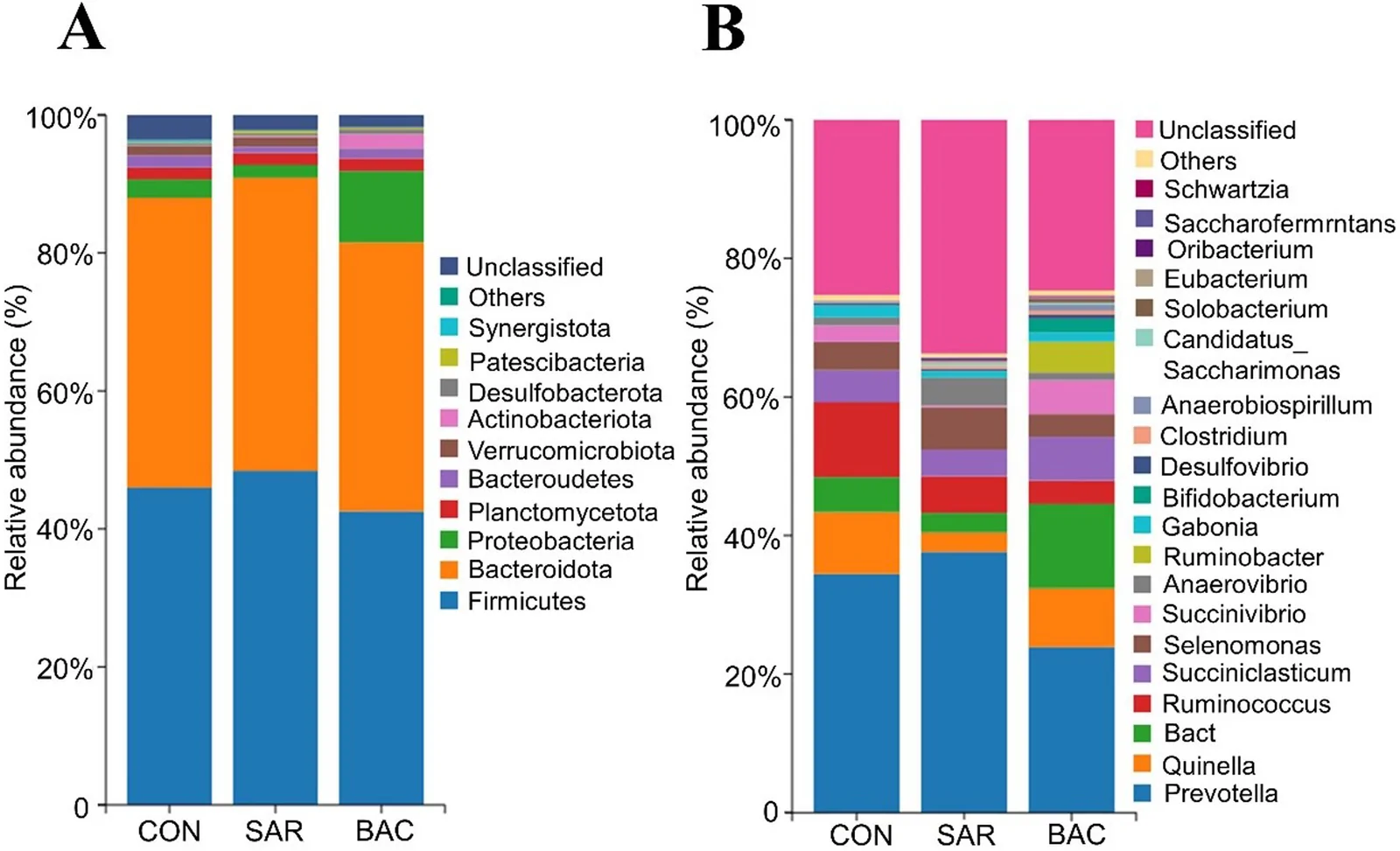
Effects of different stages on phylum and genera level composition of ruminal microbiome. CON= control stage (fed basal diet); SAR= SARA induction stage (fed high barley diet); BAC= bacteriocin feeding stage (fed high barley diet with 500 mg/kg polymyxin CP7). Different colors represent different phylum or genera.

**Fig. 5:**
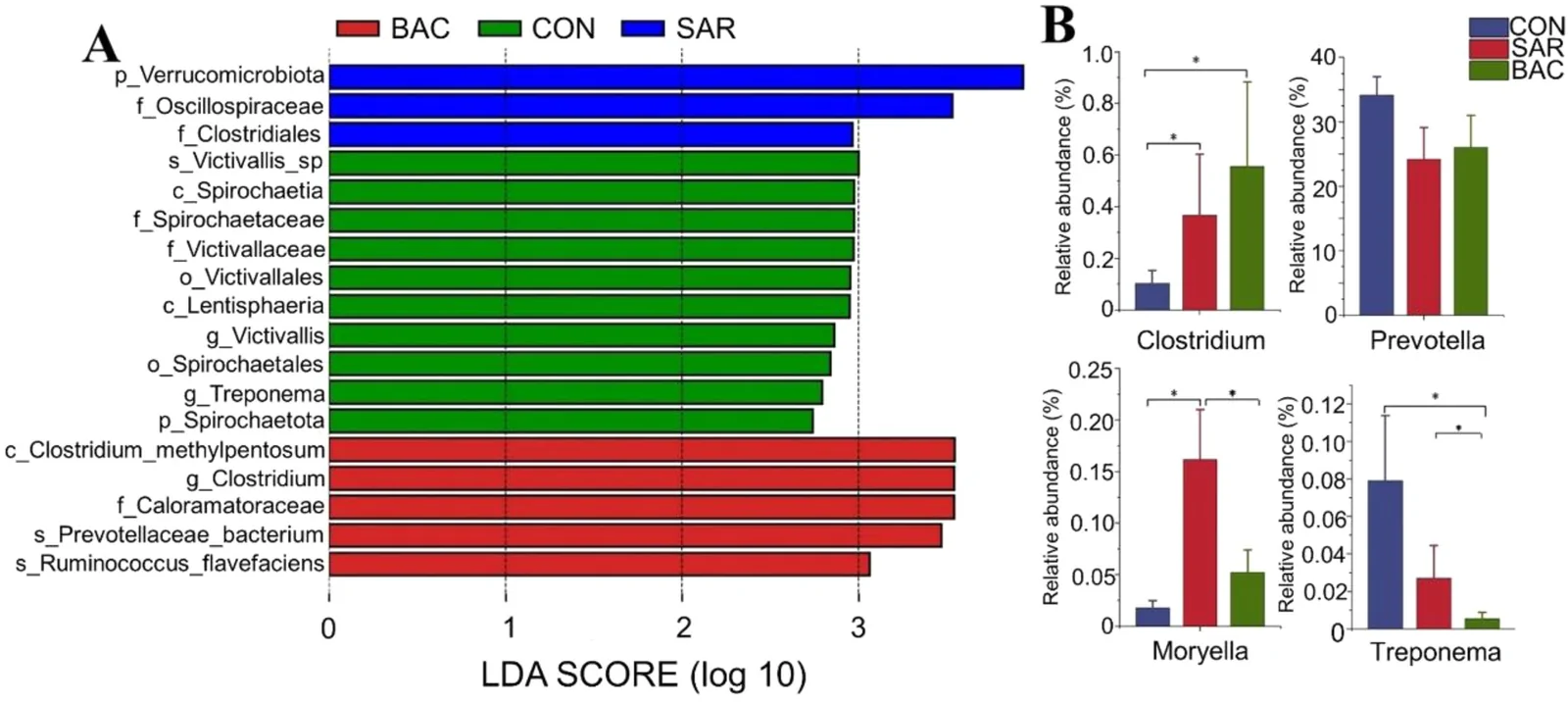
Effects of different stages on the featured taxon of *Hu* lambs. A. Selected taxa based on LEfSe analysis. B. Relative abundance of the genus *Clostridium, Prevotella, Moryella* and *Treponema*. CON= control stage (fed basal diet); SAR= SARA induction stage (fed high barley diet); BAC= bacteriocin feeding stage (fed high barley diet with 500 mg/kg polymyxin CP7). *P < 0.05.

## 4 Discussion

The subacute ruminal acidosis is usually characterized by abnormal and intermittent drops in rumen pH (Villot et al., 2018). In our study, the rumen pH dropped to less than 5.60 for 5–8 h after consuming a barley-rich diet, which indicated the sheep experience SARA. The hosts experience SARA often develop complications or other diseases and are physiologically associated with immunosuppression and inflammation (Sato, 2015). Under normal physiological conditions, the rumen contain a small amount of LPS, which is harmless to animal health. However, during SARA, rumen free LPS increased and transferred into the blood circulation, activating the inflammatory response (Sato, 2015). In the present study, the concentration of LPS in the rumen was the highest 8 h after consuming a high barley grain diet, indicating that the lower rumen pH caused a large number of Gram-negative bacteria to perish, and LPS accumulated in the rumen as residues of bacterial lysis. This result was consistent with the short-term induction of SARA in heifers by Kent-Dennis et al. (2019) and the change of LPS concentration in the rumen during the short-term transition from a high coarse feed diet to a high concentrate diet in rumen-fistulated Jersey steers as reported by Gozho et al. (2006). Moreover, high grain dietary intake in ruminants may increase the proliferation and disintegration rate of bacteria, resulting in the production of free LPS during SARA (Plaizier et al., 2014). The increase of LPS concentration in rumen activates the NF-κB and MAPK signaling pathways, leading to the release of inflammatory cytokines in rumen epithelium, causing rumen gastritis (Zhao et al., 2018). Polymyxin CP7, as an antibacterial substance that specifically inhibits Gram-negative bacteria, can maintain the normal function of the gastrointestinal tract and balance the disturbance of gut microorganisms (Soni et al., 2021). Kumar and Shin (2020) studied antimicrobial peptides that can combat bacterial infection and sepsis and found that duck cathelicidin, as a kind of poultry antimicrobial peptide, exhibits strong bactericidal activity. In the present study, the lower ruminal LPS concentration observed during the BAC stage may be partly attributed to the well-established ability of polymyxins to bind the lipid A component of LPS (Velkov et al., 2010), as well as to alterations in the ruminal microbial community. However, because the BAC stage followed the SAR stage without a concurrent SARA control receiving the same high-barley diet but no CP7, a contribution from time-dependent adaptation to the prolonged high-barley challenge cannot be excluded. Importantly, polymyxin CP7 did not restore ruminal pH during the BAC stage, and the acidosis index remained elevated. Therefore, CP7 should not be considered to have resolved the underlying ruminal acidosis. Rather, the lower ruminal LPS concentration and changes in nutrient digestibility, fermentation characteristics, and microbial composition observed during the BAC stage suggest that CP7 may influence selected consequences associated with SARA while the acidotic challenge persists.

Due to the high rumen degradable starch content in the barley diet, the rumen fermentation tended to produce more propionate (Johnson et al., 2020). The propionate can synthesize glucose through gluconogenic pathway to provide energy for the body in ruminant (Smith et al., 2018). However, there was no differences in propionate proportion and TVFA concentration in the rumen in SARA induction stage in our study, which was inconsistent to the results of Giger-Reverdin et al. (2014). The higher propionate proportion observed during the BAC stage may be partly related to the reported effects of antimicrobial peptides on ruminal fermentation (Ren et al., 2019). However, period effects, dietary adaptation, and residual effects of the preceding SAR stage may also have contributed to this response. Analogously, the study of Hino et al. (1993) showed that 12.5–25 mg/L of aibellin enhanced propionate production without significantly affecting production of total VFA, protozoal survival, or cellulose digestion. Isomeric short-chain fatty acids are mainly synthesized by branched-chain amino acids and are the main nutrients that promote the growth of rumen cellulolytic bacteria (Liu et al., 2016). The decrease in ruminal pH may suppress cellulolytic bacteria, while increasing the availability of branched-chain fatty acids, such as isobutyrate and isovalerate. This may partly explain the higher proportions of isobutyrate and isovalerate observed during the SAR and BAC stages. The lactate acidosis theory proposed by Krause and Oetzel (2005) suggested that lactic acid accumulation in the rumen was also one of the mechanisms of acidosis. Rabaza et al. (2020) believe that feeding high-grain diets to cattle can increase lactic acid concentration in rumen that cause SARA. Ruminal lactic acid accumulation depends on the balance between lactate production and utilization by the microbial community (Krause & Oetzel, 2006). Therefore, the lower lactate concentration during the BAC stage may reflect alterations in this microbial metabolic balance.

The results of this study showed that there was no difference in DMI between SAR stage and CON stage. The results of Li et al. (2017) and Aditya et al. (2017) were in accordance to our study. Zotti et al. (2017) added monensin to the Nellore cattle diet and found the DMI increased, and reduce the risk of SARA. DMI was lower in the BAC stage than in the CON stage, whereas no significant difference was observed between the SAR and BAC stages. Given the sequential nature of the experimental design, the lower DMI observed during the BAC stage cannot be attributed specifically to polymyxin CP7. It may also reflect period-related effects, prolonged exposure to the high-barley diet, physiological adaptation, or cumulative effects of the preceding SARA challenge. Because treatment and period were inherently confounded in the present design, these possibilities could not be fully distinguished. The apparent digestibility of OM and NDF was higher during the BAC stage than during the CON and SAR stages. One possible explanation is that polymyxin CP7 may influence fiber degradation through changes in cell wall-associated lytic enzyme activity (Le et al., 2017), although these enzyme activities were not directly measured in the present study.

As an immunomodulator, bacteriocin not only inhibit the secretion of pro-inflammatory cytokines, but also regulate the adaptive immune response of T lymphocytes (Lai and Gallo, 2009). Rodríguez-Lecompte et al. (2014) found that the number of WBC in cows with SARA did not change significantly, which was consistent with the results of this study. Several hematological variables differed among the experimental stages. RBC-He and MCV were lower during the SAR stage than during the CON and BAC stages, whereas MCHC, PLT, and PCT were higher during the SAR stage. These changes suggest that the SARA challenge was accompanied by alterations in erythrocyte- and platelet-related hematological profiles. Notably, these indices during the BAC stage were comparable to those during the CON stage, while the MONO proportion was higher during the BAC stage than during the CON and SAR stages. However, these hematological changes alone are insufficient to indicate specific pathological conditions or functional changes in circulating immune cells.

Gastrointestinal microbial diversity is the result of active selection and co-evolution of hosts in vivo (Bäckhed et al., 2005). Studies have shown that the addition of *Paenibacillus polymyxa* 10 to the diet has no effects on the alpha and beta diversity of the cecum of broilers (Wang et al., 2021). Although no significant differences were observed in alpha-diversity indices among the three stages, beta-diversity analysis revealed distinct microbial community structures. These findings suggest that the dietary challenge and subsequent CP7 supplementation were associated primarily with changes in community composition rather than a loss or gain of overall microbial richness and evenness. In particular, the relative abundance of *Moryella* was higher during the SAR stage than during the CON and BAC stages, suggesting that this genus was associated with the microbial community shift occurring during SARA. *Moryella*, a member of the *Lachnospira* family of *Firmicutes*, can produce SCFA through the fermentation of dietary polysaccharides (Laffin et al., 2019). Corresponding to the above, rumen butyrate increased in SAR stage in our study. The relative abundance of *Treponema* was lower during the BAC stage than during the CON and SAR stages. Liu et al. (2017) also obtained the same results by adding antimicrobial peptides to the diet of juvenile goats. Although some members of *Treponema* have been associated with pathogenic conditions, the genus is functionally heterogeneous (Choi et al., 1997), and ruminal *Treponema* species may also participate in the degradation of plant polysaccharides and fiber. Species-level or functional analyses would be required to determine whether this change contributed to the observed alterations in ruminal fermentation and nutrient digestibility. The relative abundance of *Clostridium* also differed among the experimental stages. Some members of *Clostridium* are involved in carbohydrate degradation and fermentation (Desvaux, 2006), suggesting that the observed change may reflect alterations in the ruminal fermentation environment. However, given the considerable functional diversity within this genus, its specific contribution to ruminal fermentation cannot be determined from the taxonomic resolution of the present study.

Several limitations should be considered when interpreting the present findings. The sequential design used in the present study should be considered when interpreting the BAC-stage responses. Although this design was selected to minimize potential carry-over effects associated with the incomplete reversibility of experimentally induced SARA and has been adopted in previous SARA intervention studies (Guo et al., 2013), all sheep underwent the CON, SAR, and BAC stages in the same order. Consequently, experimental stage was inherently confounded with chronological period, and the statistical analysis could not completely separate CP7-associated responses from period effects, adaptation to prolonged high-barley feeding, or residual effects of the preceding SAR stage. Future studies using parallel treatment groups or other designs capable of separating treatment and period effects are warranted to confirm these findings. Second, the relatively small sample size (n= 8) may limit statistical power and the generalizability of the findings, particularly given the number of physiological and microbiological variables evaluated. The microbiota findings should therefore be considered exploratory and require confirmation in larger, independent animal populations. Furthermore, the genus-level resolution of the 16S rRNA gene sequencing data limits species-specific and functional interpretation.

## 5 Conclusions

In conclusion, experimentally induced SARA was characterized by sustained ruminal pH depression, increased ruminal LPS concentration, and alterations in ruminal fermentation, hematological variables, and microbial composition. Although ruminal acidosis persisted during the BAC stage, lower ruminal LPS concentration, higher apparent digestibility of OM and NDF, and alterations in ruminal fermentation and microbial composition were observed compared with the SAR stage. These findings suggest that polymyxin CP7 may modulate selected responses associated with SARA rather than correct the underlying ruminal acidosis. However, given the sequential experimental design, potential period, adaptation, and residual effects cannot be fully separated from CP7-associated responses and should be considered when interpreting these findings.

## Ethics declaration

This study was conducted in accordance with the ARRIVE (Animal Research: Reporting of In Vivo Experiments) guidelines.

This study was approved by the Animal Ethics Committee of Lanzhou University.

## Funding

This work was supported by the Science and Technology Planning Project of Gansu Province (26ZDNG002), the Central Guidance for Local Science and Technology Development Fund Project (25ZYJA022) and the Tianchi Talent Introduction Program of Xinjiang Uygur Autonomous Regions.

## CRediT authorship contribution statement

**Shuai Jiao:** Writing – review & editing, Writing – original draft, Methodology, Data curation, Conceptualization. **Baozhen Zhu:** Formal analysis, Data curation. **Fei Li:** Writing – review & editing, Funding acquisition, Conceptualization. **Zhiyuan Ma:** Validation, Investigation. **Fadi Li:** Supervision, Conceptualization. **Xiuxiu Weng:** Supervision, Conceptualization. **Ying Yu:** Supervision, Resources. **Ping Li:** Supervision, Resources. **Rui Xu:** Writing – review & editing, Investigation.

## Declaration of competing interest

The authors hereby declare that they do not have any known competing financial interests or personal relationships that might have influenced the research presented in this paper.

## Data Availability

The sequencing data obtained in this study were deposited in the NCBI Sequence Read Archive (SRA) under accession numbers SRR28034552 to SRR28034566.
